# Non‑Hodgkin's lymphomas of the lacrimal sac: Current insights and future directions (Review)

**DOI:** 10.3892/mi.2024.167

**Published:** 2024-06-10

**Authors:** Michail Athanasopoulos, Georgios Nomikos, Pinelopi Samara, Stylianos Mastronikolis, Christos Tsilivigkos, Nicholas S. Mastronikolis

**Affiliations:** 1 Department of Otolaryngology, University Hospital of Patras, 26504 Patras, Greece; 2 Department of Otolaryngology, General Hospital of Nikaia, Piraeus ‘Agios Panteleimon’, 18454 Athens, Greece; 3 Children's Oncology Unit Marianna V. Vardinoyannis-ELPIDA, Aghia Sophia Children's Hospital, 11527 Athens, Greece; 4 Department of Ophthalmology, Medical School, University of Patras, 26504 Patras, Greece; 5 1st Department of Otolaryngology, Hippocrateion Hospital, National and Kapodistrian University of Athens, 11527 Athens, Greece

**Keywords:** lacrimal sac, non-Hodgkin's lymphoma, chemotherapy, radiotherapy, immunotherapy

## Abstract

Non-Hodgkin's lymphoma (NHL) of the lacrimal sac is a rare, yet clinically significant entity within the spectrum of ocular malignancies. While primary lacrimal sac lymphoma is uncommon, it poses unique diagnostic and therapeutic challenges due to its anatomical location and potential for aggressive behavior. Despite advancements being made in the current understanding and treatment of NHL, research that specifically addresses the involvement of the lacrimal sac is currently lacking. Thus, the present review aimed to provide insight into the epidemiology, clinical presentation, diagnostic modalities, histopathological features, treatment strategies and prognosis of lacrimal sac NHL. Through a methodical analysis of previous literature, the present review highlights the diverse spectrum of NHL subtypes that affect the lacrimal sac, including diffuse large B-cell lymphoma, extranodal marginal zone lymphoma, mantle cell lymphoma and follicular lymphoma. Moreover, the present review discusses the role of advanced imaging techniques in accurate staging and treatment planning, including computed tomography (CT), magnetic resonance imaging and positron emission tomography-CT. The present review also discusses evolving treatment approaches, such as surgical intervention, chemotherapy, radiotherapy, immunotherapy, combinations of the aforementioned treatments and targeted therapy. In addition, the present review highlights the significance of multidisciplinary collaboration in attaining optimal outcomes for individuals with lacrimal sac NHL. The present review aimed to provide a basis for 'further investigations into novel treatment modalities and prognostic markers that may aid in guiding personalized management strategies, ultimately improving outcomes for patients with NHL.

## 1. Introduction

Non-Hodgkin's lymphoma (NHL) includes a diverse group of diseases originating from lymphoid tissues, encompassing a wide range of histological subtypes and clinical presentations. In 85-90% of cases, NHL arises from B-cells, with less frequent occurrences from natural killer and T-cells. The pathogenesis of NHL is multifactorial, involving genetic, environmental and immunological factors. Chromosomal translocations, gene mutations and dysregulated signaling pathways, such as the B-cell receptor pathway, the NF-κB pathway, and the PI3K-AKT-mTOR pathway, contribute to the overall development of NHL. These alterations disrupt normal cellular processes, leading to uncontrolled proliferation and the survival of malignant lymphocytes (1).

As one of the most prevalent hematological conditions worldwide, NHL poses significant diagnostic and therapeutic challenges due to its heterogeneous nature and variable clinical courses. NHL accounts for 3-4% of all cancer cases annually, with notable geographic and ethnic variations in incidence, placing it among the top ten most common malignancies in developed countries. The overall incidence of NHL has continued to increase over the past few decades, partly due to improved diagnostic techniques and changes in risk factors (2). Moreover, NHL-associated deaths account for ~2.5% of all cancer-related deaths (3). While NHL commonly affects the lymph nodes, extranodal involvement may occur in various organs and tissues throughout the body, including the ocular adnexa. Among these extranodal sites, the lacrimal sac represents a rare, yet clinically significant location for NHL development (4).

**Figure 1:**
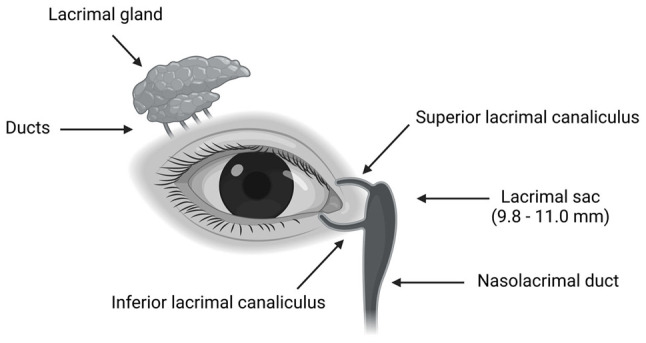
Illustration depicting the anatomy of the efferent lacrimal system, including the lacrimal canaliculi, lacrimal sac, nasolacrimal duct, and associated structures. This system facilitates the drainage of tears from the ocular surface into the nasal cavity (the figure was created using BioRender.com).

The lacrimal sac, located in the medial canthal region of the orbit, is crucial for tear drainage into the nasolacrimal duct. Positioned within the anteromedial orbit wall, it resides within a concave fossa bordered by the anterior and posterior lacrimal crests, separated by the lacrimomaxillary suture (5). Notably, there are histological similarities with the nasolacrimal duct, and the conical portion within this fossa is identified as the lacrimal sac. Encased laterally by the lacrimal fascia and posteriorly by the common fascia of Horner's muscle, it forms the lacrimal diaphragm (6). The sac measures 9.8-11.0 mm in height, 7.5 mm in anteroposterior diameter and 3.0-4.9 mm in horizontal diameter, featuring a lining composed of stratified columnar epithelium with goblet cells, serous glands and cilia (7). Elastic fibers within the lamina propria aid in its function as a pump (8), while the submucosa contains lacrimal drainage-associated lymphoid tissue, contributing to the defense of the nasolacrimal system (9) (Fig. 1).

Primary NHL of the lacrimal sac is uncommon, with the majority of cases arising secondary to systemic lymphoproliferative disorders (10). NHL subtypes that affect the lacrimal sac include diffuse large B-cell lymphoma (DLBCL), extranodal marginal zone lymphoma (MZL), mantle cell lymphoma (MCL) and follicular lymphoma (FL). Due to its proximity to the nasal cavity, the lacrimal sac is vulnerable to mucosa-associated lymphoid tissue (MALT) lymphoma, a subset of extranodal MZL. Despite its small size, the lacrimal sac accumulates lymphoid tissue and participates in immune surveillance, predisposing it to MALT lymphoma (11). The clinical presentation of lacrimal sac NHL is often indicative of a benign condition, such as dacryocystitis or nasolacrimal duct obstruction, leading to complexities in diagnosis and delayed identification. In addition, the effective management of lacrimal sac NHL requires a multidisciplinary approach, involving ophthalmologists, otolaryngologists, oncologists, pathologists and radiation oncologists, to ensure accuracy in diagnosis, staging and treatment.

The present review aimed to provide a comprehensive overview of lacrimal sac NHL, encapsulating the epidemiology, clinical manifestations, diagnostic modalities, histopathological features, treatment strategies and prognosis. Through the analysis of previous literature and clinical experience, the present review aimed to provide current insight into this disease, and determine potential future investigations to improve patient outcomes.

## 2. Epidemiology and classification

### The incidence and prevalence of lacrimal sac NHL

Lacrimal sac tumors are uncommon in medical practice. In total, ~55% of these tumors are malignant, with ~71% originating from epithelial cells (12). Among lacrimal sac tumors, lymphomas account for 2-8% (13). These lymphomas are often secondary occurrences due to lymphatic metastasis from other regions, with primary lacrimal lymphomas being particularly uncommon. Notably, primary lacrimal sac lymphomas often manifest in older individuals, affecting a patient population with a median age of 71 years (range, 45-95 years). In addition, ~80% of affected patients are >60 years of age (14). Therefore, the majority of documented cases arise within the elderly demographic, with a notable predominance among females.

NHL occurrences in pediatric patients are infrequent, as the median ages of patients affected by DLBCL and MALT lymphomas are ~70 and ~60 years, respectively. Lacrimal sac NHL is exceptionally rare in children. To date, few pediatric cases of primary NHL of the lacrimal drainage system have been reported. Previous reports have detailed the cases of a poorly differentiated lymphoma (15), MALT lymphoma (16-18) and DLBCL (19-21).

### Subtypes, classification and staging of lacrimal sac NHL

The main subtypes encountered in lacrimal sac NHL include DLBCL, MALT lymphoma, MCL and FL. Briefly, DLBCL is characterized by a diffuse growth pattern of large B-cells with enlarged nuclei. However, various subtypes of this lymphoma have been identified, each with unique morphological, biological and clinical features. MALT lymphoma often exhibits a low grade; however, this may evolve into DLBCL. In addition, DLBCL exhibits an indolent nature. MCL originates from B-cells in the mantle zone of lymphoid follicles and is often associated with the t(11;14) (q13;q32) chromosomal abnormality, resulting in the upregulation of cyclin D1. FL arises from germinal center B-cells and typically presents with follicular growth patterns. It is often characterized by the t(14;18) (q32;q21) chromosomal translocation, leading to the upregulation of BCL2. Other rare subtypes of lacrimal sac NHL include peripheral T-cell lymphoma, Burkitt's lymphoma and lymphoplasmacytic lymphoma (22).

Classifications are based on morphology, phenotype, cytology, genetics and molecular features, along with clinical characteristics, etiology and pathogenesis. Tumor classification is carried out to define the plethora of subtypes based on cellular origin. Over the years, lymphoma staging systems have evolved, incorporating criteria for therapy response assessment with computed tomography (CT) and positron emission tomography (PET)-CT. The Lugano classification, introduced in 2014, standardizes staging and response evaluation for both Hodgkin's lymphoma and NHL, building on the previous Ann Arbor system that originated in the 1970s (Table I) (23).

Various stages of NHL are subdivided into categories denoted by letters; namely, A, B, E, S and X, depending on the presence or absence of symptoms in affected individuals. Briefly, category A signifies the absence of systemic symptoms at the time of diagnosis, category B indicates the presence of B symptoms (night sweats, unexplained fever and/or unexplained weight loss), category E indicates that the malignancy affects a single organ outside the lymphatic system or has extended from a lymph node to another organ, category S signifies the involvement of the spleen, and category X denotes the presence of large masses of lymphoma (bulky disease).

While the Lugano/Ann Arbor staging systems rely on imaging techniques along with the presence or absence of B symptoms, they fall short in precisely locating lacrimal sac tumors and determining their extension into neighboring structures. This limitation is effectively addressed by the Tumor, Node, Metastasis (TNM) staging system provided by the 8th edition of The American Joint Committee on Cancer. This system categorizes primary tumor location and regional extension (T), lymph node involvement and severity (N), and the presence or absence of distant involvement of extranodal organs outside of the ocular adnexal region (M), including the bone marrow (24).

## 3. Clinical presentation: Signs and symptoms of lacrimal sac NHL

In the early stages of NHL, patients may not experience symptoms. However, the majority of patients with NHL typically present with concurrent lymphadenopathy and potential B symptoms. Notably, pain is not commonly reported. Additional symptoms may manifest depending on the organ involved (25).

When the lacrimal sac is affected, specific symptoms often include epiphora, persistent edema or a mass in the medial canthal region, redness or irritation of the eye, discharge from the inner corner of the eye (clear, mucoid or purulent), recurrent eye infections, pain or discomfort around the eye and eyelid ptosis (26). Occasionally, initial symptoms may manifest as acute dacryocystitis (27) or individuals may experience recurrent episodes of dacryocystitis (28). In the case that lacrimal sac NHL extends into adjacent structures of the eye or the nose, or involves blood vessels within the lacrimal sac area, additional symptoms may be experienced by patients. These symptoms may include decreased visual accuracy, diplopia/visual disturbances, proptosis/displacement of the eye, retrobulbar resistance or chemosis, as well as symptoms affecting the nasal cavity, such as sinusitis, nasal obstruction and epistaxis (Table II) (29).

The diagnosis of lacrimal sac tumors may be challenging. In the absence of a palpable mass, experienced ophthalmologists may negate various factors. Lymphoma may originate from lacrimal drainage-associated lymphoid tissue, potentially causing epiphora without a palpable mass in the early stages (30). Dacryocystography (DCG) is used to reveal a filling defect indicative of a lacrimal sac tumor (31), while blood-stained discharge is often indicative of advanced disease stages. A palpable mass or edema, particularly above the medial canthal tendon, is a key indicator of a lacrimal tumor.

## 4. Differential diagnosis from alternate lacrimal sac pathologies

Conditions affecting the lacrimal sac exhibit considerable variation, yet their symptomatology often remains consistent across cases. A diverse range of tumor types may occur within this system, broadly classified into three groups; namely, primary epithelial, primary non-epithelial and inflammatory lesions. Primary epithelial tumors commonly include squamous cell carcinoma, papilloma and transitional cell carcinoma. Primary non-epithelial tumors include malignant lymphoma, fibrous histiocytoma and malignant melanoma. Inflammatory lesions often consist of sarcoidosis, Wegener granulomatosis and pyogenic granuloma (32).

Given the nature of lacrimal sac pathologies, a comprehensive medical history and thorough physical examination are essential for an accurate diagnosis. Ancillary diagnostic tools, such as DCG, CT and magnetic resonance imaging (MRI), are crucial in guiding diagnosis and for treatment planning by providing detailed anatomical information (33). However, an incisional biopsy of the lesion for histopathological and cytological examination remains the gold standard for a definitive diagnosis, particularly in suspected tumor cases (34). This approach is crucial for guiding the formulation of the most appropriate treatment strategy.

## 5. The importance of early detection and diagnosis in lacrimal sac NHL

The early detection and diagnosis of lacrimal sac NHL are crucial for optimizing treatment outcomes, preserving the quality of life and improving the overall prognosis of patients. Timely intervention enables healthcare providers to develop personalized treatment plans, prevent disease progression and minimize complications, such as local invasion, the requirement for reconstruction and metastasis to distant sites. With early diagnosis, patients may have a higher chance of achieving complete remission and may experience fewer symptoms. Moreover, early detection may reduce healthcare costs associated with aggressive late-stage treatments, and provide patients and their families with timely counseling, improving their overall understanding of the condition. Therefore, regular eye examinations and the prompt evaluation of any concerning symptoms are essential for individuals who are at risk of developing lacrimal sac NHL, such as those with a history of chronic dacryocystitis, previous radiation therapy to the head and neck, immunodeficiency disorders, or a family history of lymphoproliferative diseases (2).

## 6. Prognostic assessment for NHL: The role of the International Prognostic Index (IPI)

The prognosis of lacrimal sac NHL is dependent on tumor type, malignancy, disease stage, tumor grade and the overall health of the patient. IPI is a useful tool for outcome predictions, as stated by the International Non-Hodgkin's Lymphoma Prognostic Factors Project 1993(35). This project accounts for several factors, including age (as patients >60 years of age may exhibit a poorer prognosis), performance status (as a poorer performance status may be indicative of a worse prognosis), stage of disease, serum lactate dehydrogenase (LDH) levels, with elevations associated with a poorer prognosis, and number of extranodal sites involved, as the involvement of multiple extranodal sites may be indicative of a worse prognosis. Each of these factors is assigned a score, and the total score is used to categorize patients into low-, intermediate- or high-risk groups (35). Patient grouping aids clinicians in determining appropriate treatment strategies and predicting patient outcomes.

The IPI was structured around patients receiving cyclophosphamide, hydroxydaunorubicin, Oncovin (vincristine) and prednisone (CHOP) chemotherapy. Notably, the addition of the chimeric anti-CD20 monoclonal antibody, rituximab, to traditional CHOP chemotherapy (R-CHOP) may be used in some patients (36). The restructuring of the IPI parameters into a revised version (R-IPI) yields a more clinically insightful prognosis. The R-IPI stratifies patients into three distinct prognostic groups, namely, very favorable [4-year progression-free survival (PFS), 94%; overall survival (OS), 94%]; favorable (4-year PFS, 80%; OS, 79%); and unfavorable (4-year PFS, 53%; OS, 55%) outcomes (37).

## 7. Pathogenesis and risk factors of lacrimal sac NHL

The pathogenesis of lacrimal sac NHL is characterized by a dysregulated lymphocyte proliferation within the lacrimal sac, culminating in tumor formation. While the precise etiology remains elusive, a complex interplay of factors contributes to its development. Immunodeficiency, stemming from conditions, such as HIV/AIDS (38) or post-organ transplantation immunosuppression (39), creates a conducive environment for lymphoma emergence. Chronic inflammation, often associated with conditions such as chronic dacryocystitis, may exacerbate lymphocyte activation and proliferation, thus promoting tumorigenesis (40). Infectious agents, including Epstein-Barr virus and human herpesvirus 8, are implicated in the pathogenesis of NHL, with the potential to direct stimulate lymphocyte proliferation and malignant transformation (41).

Genetic predisposition also plays a role, with alterations in lymphocyte regulatory genes promoting uncontrolled cell growth and tumor initiation. Notably, various genetic susceptibility loci for DLBCL have been identified through genome-wide association studies, and these have highlighted the role of pathways associated with immune recognition and immune function (42). Moreover, environmental factors, such as exposure to certain toxins or chemicals, may contribute to the development of NHL; however, this notion remains to be fully elucidated.

Risk factors, such as advanced age, may increase the susceptibility to lacrimal sac NHL development, due to immunosenescence and accumulated genetic mutations. Females also exhibit a higher predisposition to this disease. In addition, individuals with a previous history of lymphoma or other hematologic malignancies may also be at an increased risk, as a result of hormonal factors and potential shared genetic predispositions. Autoimmune diseases, such as Sjögren's syndrome, which involve chronic inflammation and immune dysregulation, further increase the susceptibility to lacrimal sac NHL development (43). In addition, systemic immunosuppression and certain medications may compromise immune surveillance, promoting lymphoma development. Occupational exposures with potential carcinogenic agents, such as pesticides, solvents, dyes, engine exhaust, wood dust, wood finishing chemicals and various microbes, are also associated with an increased risk of this disease. The identification of specific agents remains challenging (44).

Overall, the pathogenesis and risk factors for lacrimal sac NHL are multifaceted, encompassing a dynamic interplay of immunological, environmental, genetic and infectious elements. Understanding these complex mechanisms is crucial for elucidating disease progression and developing targeted therapeutic interventions.

## 8. Prognosis, long-term outcomes and survival rates of patients with lacrimal sac NHL

Numerous factors have been identified as independent predictive indicators of a poor OS in cases of ocular NHL. Factors include a patient age of >59, elevated LDH levels, advanced tumor stage, high-grade histological subgroups and the presence of B symptoms. Specifically, in ocular adnexal DLBCL, concurrent bone marrow involvement is recognized as an independent adverse prognostic factor (45). Furthermore, p53 expression levels are associated with the presence of the Ki-67 antigen, and this association is indicative of clinical outcomes (46).

Disease localization may affect the survival rates of patients, with a notably high 5-year survival rate of 90.9% when the disease is confined to one area. However, prognosis is less favorable in patients with advanced-stage disease, with a 5-year survival rate of 23.5% (47). In the broader category of lacrimal sac lymphomas, the overall 5-year survival rate is 65% (14). Notably, female patients with lymphoma affecting the lacrimal drainage system may experience more pronounced symptoms and demonstrate a heightened likelihood of disease recurrence. However, the precise association between this tumor and hormonal response remains to be established (48,49).

## 9. Diagnostic approaches

During a clinical examination, distinguishing between benign and malignant lymphoid lesions remains challenging. Thus, obtaining a tumor biopsy is imperative for accurate diagnosis and the initiation of required systemic assessments.

### Imaging studies: Challenges and advances in lacrimal sac NHL

Conventional imaging modalities, such as CT and MRI, play a crucial role in the initial assessment of lacrimal sac NHL, providing valuable information on disease location, severity and anatomical features, for accurate diagnosis and treatment planning. CT imaging is useful for detecting bony erosion, whereas MRI provides insight into the characteristics of NHL (50). However, both CT and MRI exhibit limitations in the precise characterization of lacrimal sac lesions, due to their limited soft tissue resolution. Challenges include distinguishing between benign and malignant lesions, accurate disease staging and evaluating treatment response. Moreover, the proximity of the lacrimal sac to critical structures, such as the orbit and nasal cavity, requires the precise delineation of tumor involvement.

Advanced imaging techniques, such as fluorine-18-fludeoxyglucose PET-CT and diffusion-weighted imaging, exhibit potential in improving diagnostic accuracy through acquiring functional and metabolic information. PET-CT imaging also aids in treatment response assessment and disease recurrence detection (51). Notably, interpreting imaging findings in lacrimal sac NHL requires expertise and collaboration among clinicians to ensure accurate diagnosis and optimal patient care.

Guo *et al* (52) revealed that MALT lymphomas typically exhibit homogeneous and isointense patterns on both T1WI and T2WI MRI sequences, along with mild to moderate enhancement and a plateau pattern on dynamic contrast-enhanced MRI. Notably, the results of their study demonstrated that in some cases, lacrimal sac lymphomas may lead to enlarged lacrimal ducts with bone compression, without evidence of bone erosion. These factors may be associated with the low-grade nature of the MALT lymphoma (52).

### Molecular insights into lacrimal sac NHL: Unraveling diagnostic subtypes and biomarkers with a focus on DLBCL

The molecular mechanisms underlying lacrimal sac NHL are complex and are not yet fully established due to the rarity of this condition. However, several key pathways and genetic alterations have been implicated in the pathogenesis of NHL, which may also apply to tumors affecting the lacrimal sac.

The dysregulation of B cell signaling is common, leading to uncontrolled proliferation and the survival of malignant B cells. Genetic abnormalities, including chromosomal translocations, gene mutations and copy number alterations, play a critical role in lymphomagenesis. The dysregulation of apoptosis and cell cycle control pathways contributes to the survival and proliferation of malignant lymphocytes in NHL. The tumor microenvironment, comprising immune cells, stromal cells and extracellular matrix components, plays a crucial role in the pathogenesis of NHL. Crosstalk between malignant lymphocytes and the microenvironment promotes tumor growth, immune evasion and treatment resistance. Epigenetic modifications, including DNA methylation, histone modifications and non-coding RNA dysregulation, also contribute to the pathogenesis of NHL by regulating gene expression patterns (53,54).

Molecular diagnostics are crucial for confirming the subtype of lacrimal sac NHL and identifying associated biomarkers, which are essential for accurate diagnosis and the development of personalized treatment strategies. Moreover, molecular biomarkers provide valuable prognostic insight, such as the aggressiveness of the disease and the likelihood of treatment response. Biomarkers may include gene mutations, protein expression levels, or other molecular signatures associated with disease progression and patient outcomes.

Through gene expression profiling, DLBCL is stratified into two prognostically significant subtypes; namely, germinal center (GC) B-cell-like (GCB) and activated B-cell-like (ABC) lymphoma (55). In a previous study, by examining the genomic landscape of 337 patients that were newly diagnosed with DLBCL, a simplified 38-gene algorithm identified seven distinct genetic subtypes. These subtypes, including TP53Mut, MCD-like, BN2-like, N1-like, EZB-like and ST2-like, were associated with unique clinical outcomes and biological features. Notably, the TP53Mut subtype was shown to be associated with a poor prognosis due to p53 dysregulation, while the MCD-like subtype was related to unfavorable outcomes associated with BCL2/MYC double-expression and NF-κB activation. Conversely, tbe BN2-like subtype demonstrated a favorable response within ABC-DLBCL and featured NF-κB activation (56).

Notably, dysregulated signaling pathways, including those involved in B-cell receptor signaling and immune checkpoint regulation, contribute to the tumor-promoting microenvironment in lacrimal sac NHL. Understanding this interplay may aid in identifying novel therapeutic targets and immunomodulatory strategies. Technological advances that enable the characterization of heterogeneous cell populations within tumors may provide insight into the tumor microenvironment. Incorporating such approaches into future research may lead to more effective treatments tailored to the specific molecular and immune landscape of lacrimal sac NHL.

## 10. Diagnostic significance of histopathological examination and immunohistochemistry: The analysis of DLBCL

Due to cost and limited accessibility, the implementation of molecular approaches in routine clinical practice is challenging. As an alternative, immunohistochemistry (IHC)-based algorithms have gained attention due to their user-friendly and practical application in pathology laboratories. These techniques allow pathologists to analyze tissue samples obtained through biopsy or surgical excision, providing valuable insight into the histological subtype, grade and immunophenotype of the tumor. IHC aids in the identification of specific cell markers and protein expression patterns, which ultimately leads to the classification of NHL subtypes and differentiation from other benign or malignant conditions.

Several markers are measured in NHL to aid in diagnosis, prognosis and treatment planning. Commonly assessed markers include CD20, CD3, CD5, CD10, BCL6, BCL2, Ki-67, MYC and cyclin D1. These markers aid in identifying the type of lymphoma, assessing tumor aggressiveness and guiding treatment decisions (57).

The method devised by Hans *et al* (58), which used IHC, was initially carried out for the categorization of DLBCL into GC and non-GC subtypes. This algorithm utilizes the expression of CD10, BCL6 and MUM1 proteins. This method requires only three widely available antibodies, thus leading to its widespread use compared with alternate subtyping algorithms for DLBCL. Moreover, that study demonstrated a strong association between the Hans algorithm and gene expression profiles, revealing distinct survival outcomes between the GCB and ABC DLBCL groups (58). However, subsequent investigations yielded varying results, with some affirming the prognostic value of the Hans algorithm, and others failing to identify statistically significant differences between subgroups (59).

Through the combination of imaging, clinical assessment and immunophenotypic analysis, clinicians may achieve a comprehensive understanding of the disease. Following the diagnosis of lacrimal sac NHL, patients undergo an extensive oncologic workup, which includes imaging that covers areas beyond the lacrimal sac region. Moreover, laboratory analyses and bone marrow biopsies are integral components of the diagnostic process, ensuring a thorough assessment of the extent of the disease and any associated characteristics.

## 11. Treatment modalities

**Figure 2:**
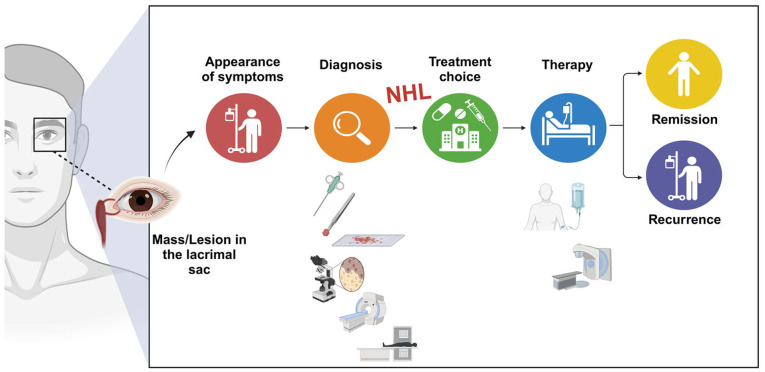
Schematic representation of the stages involved from the onset of symptoms related to the lacrimal sac, leading to the diagnosis of NHL (including biopsy, histopathology, immunochemistry and imaging), followed by the patient's comprehensive treatment plan (involving chemotherapy, immunotherapy, targeted therapy and radiotherapy) aimed at achieving complete remission and minimizing recurrence (the figure was created using BioRender.com). NHL, non-Hodgkin's lymphoma.

There are no standardized treatment protocols for the management of lacrimal sac NHL. However, several crucial factors are used to guide the initial assessment and tailor optimal treatment strategies. These factors include the following: i) the specific histopathological subtype of the lymphoma; ii) the extent of disease involvement both locally in the ocular region and systemically; iii) prognostic indicators relevant to both the patient and the disease itself; and iv) the impact of the lymphoma on visual function. Typically, a multimodal approach involving surgery, chemotherapy, radiotherapy, immunotherapy or targeted therapy is used. However, total surgical resection of orbital lymphoma is generally discouraged due to the potential risk of ocular damage and subsequent vision loss. Surgery is typically reserved for diagnostic purposes rather than therapeutic intervention. Consequently, treatment decisions are highly individualized, and these vary from one patient to another (Fig. 2).

### Surgery: Resection and/or reconstruction

Current surgical guidelines recommend the surgical removal of lacrimal sac tumors. Fine-needle aspiration biopsy remains a contentious technique compared with open biopsy, with the latter being favored in some studies. Incisional biopsy may be valuable when confirmation of a lacrimal sac tumor is difficult; thus, this process may be aided by subsequent immunohistochemical analysis for the confirmation of malignancy.

Treatment modalities vary depending on tumor type, with benign tumors typically managed through excision with dacryocystorhinostomy (DCR) or dacryocystectomy (DCT). Aggressive malignancies may require removal of the entire lacrimal drainage system, including orbital exenteration and paranasal sinus resection for extensive lesions. The total excision of the lacrimal sac (DCT without osteotomy) is the preferred approach for suspected lacrimal sac tumors, with planned modified DCR or reconstruction considered post-histological confirmation (60).

In a case series reported by Kadir *et al* (61), 60% of patients with lacrimal sac tumors initially underwent DCT with excision biopsy followed by planned modified DCR, based on tumor pathology and therapeutic response. Deep incisional biopsy was conducted in 40% of cases to ensure an accurate diagnosis. While the surgical extraction of lacrimal sac tumors is a common approach, the resection of orbital cell lymphoma is not advisable due to the elevated cure rates associated with non-surgical treatments and the potential risk of harming the eye. Therefore, extensive surgery should be avoided to maintain adequate cosmesis and preserve eye function (61). For lymphomas of the lacrimal sac, excision of the lesion with DCR or DCT is the preferred therapeutic approach.

### Chemotherapy, radiotherapy, immunotherapy and targeted therapy

Primary treatment for lacrimal sac lymphoma typically involves chemotherapy and/or radiotherapy. Chemotherapy is used in the treatment of lacrimal sac NHL, particularly in cases unresponsive to local therapies (62). Systemic chemotherapy is not recommended for patients of an advanced age, or for patients with a poor performance status or concurrent comorbidities (63).

Commonly used chemotherapeutic regimens include CHOP, cyclophosphamide, etoposide, vincristine, and prednisone (CEOP), cyclophosphamide, vincristine and prednisone (COP/CVP), cyclophosphamide, vincristine, procarbazine and prednisone (C-MOPP) and chlorambucil, and each regimen is tailored to the specific condition and response of the patient (64). The combination of chemotherapy and immunotherapy, particularly rituximab, exhibits potential in the management of lacrimal sac NHL. Rituximab has demonstrated efficacy either as a monotherapy or in combination with chemotherapy. In a recent study, notable response rates were observed in 27% of patients who received rituximab (65).

The effective management of aggressive NHL in elderly and unfit patients is challenging due to the cardiotoxicity associated with anthracyclines, despite their proven therapeutic efficacy in this lymphoma subtype. The rituximab, cyclophosphamide, oncovin (vincristine), methotrexate, and prednisone (R-COMP) regimen is comparable to rituximab-CHOP (R-CHOP); however, this regimen uses 50 mg/m^2^ Myocet in the place of doxorubicin. Notable decreases or low occurrences of severe cardiac adverse events have been observed in patients treated with R-COMP (66). Rigacci *et al* (66) conducted an in-depth retrospective analysis of 946 patients with DLBCL who received R-COMP. All included patients were aged >65 years and/or presented with underlying cardiopathy. The findings of that study validated the effectiveness of the R-COMP regimen, with a reported 3-year OS rate of 70%. These results were comparable with those obtained using R-CHOP. Notably, the incidence of cardiotoxicity was markedly low, with only 5% of patients experiencing grade 3-4 adverse events (66). These results indicated that non-pegylated liposomal doxorubicin retains therapeutic effectiveness while mitigating adverse effects.

In addition, research has focused on novel agents, such as ibrutinib, an irreversible Bruton's tyrosine kinase inhibitor. Although initially approved for chronic lymphocytic leukemia/small lymphocytic lymphoma and relapsed/refractory MCL, ibrutinib demonstrated potential in other B-cell lymphomas, including those affecting the lacrimal sac. The results of a previous study demonstrated the efficacy of ibrutinib in patients with ABC DLBCL, with an overall response rate of 37%. Moreover, the combination of ibrutinib with R-CHOP was safe in patients with untreated B-cell lymphoma (67). While chemotherapy remains fundamental in the treatment of lacrimal sac NHL, combining it with immunotherapy/targeted therapy, such as rituximab or ibrutinib, exhibits potential in improving therapeutic outcomes and patient prognosis.

Radiotherapy exhibits potential as the ideal treatment modality for both slow-progressing orbital lymphomas and aggressive NHL, with high local control rates and minimal toxicity. Recommended radiation doses vary based on the location of NHL and histological type, with typical doses ranging from 30 to 45 Gy for localized lacrimal sac lymphomas (68). Combining short-course chemotherapy with radiotherapy may exhibit potential in obtaining optimal cure rates with reduced toxicity.

External-beam radiation therapy (EBRT) is effective in the treatment of localized low-grade ocular adnexal lymphoma, with the potential for achieving local disease control rates of up to 100%, and low recurrence rates that do not exceed 15%. In 50% of the patients studied, Fernández *et al* (65) demonstrated that EBRT exhibited potential as the primary therapeutic approach, highlighting the effectiveness in this disease. The results of previous studies demonstrated high local control rates with EBRT, including a 7-year local control rate of 97% in patients with localized MALT lymphoma treated with a dose of 25 Gy (69), and a 5-year local control rate of 95% in patients treated with doses of 24 to 25.5 Gy (70). In addition, palliative or re-irradiation doses of 4 Gy yielded a complete response rate of ~85% in patients with indolent ocular adnexal lymphoma (71). Collectively, these results highlight the effectiveness of radiotherapy in achieving disease control while minimizing treatment-associated adverse events.

Further research and clinical trials that specifically focus on ocular NHL are required, in order to fully elucidate the efficacy and potential of these innovative treatment modalities in effectively managing this condition.

## 12. Gaps in current knowledge and future perspectives

### Challenges in research, unanswered questions and areas requiring further investigation

Research in lacrimal sac NHL faces several challenges, primarily due to the rarity of this malignancy. The limited availability of tissue samples and insufficient data on disease mechanisms impact the understanding and development of targeted therapeutic strategies. In addition, a lack of standardized diagnostic criteria and treatment protocols may lead to variability in study outcomes and limited comparability between studies.

Despite advances, several unanswered questions remain regarding lacrimal sac NHL. Key areas requiring further investigation include elucidating the exact molecular pathways underlying disease development and progression, identifying novel biomarkers for early detection and prognosis, and optimizing treatment approaches to improve outcomes and reduce treatment-related toxicities. In addition, further investigations are required to focus on the role of immune dysregulation, environmental factors and genetic predisposition in the disease pathogenesis.

### Implications for personalized medicine

The emergence of personalized medicine exhibits potential in the treatment of lacrimal sac NHL. Through the integration of genomic profiling, molecular characterization and clinical data, personalized treatment strategies can be tailored to individual patients, optimizing therapeutic efficacy while minimizing adverse effects. Moreover, advancements in precision medicine may facilitate the development of targeted therapies, immunotherapies and novel treatment modalities specifically designed to target the unique molecular signatures of lacrimal sac NHL subtypes. Collaborative efforts among researchers, clinicians and industry stakeholders are essential for translating these insights into clinical practice, ultimately improving patient outcomes.

## 13. Conclusions

In conclusion, lacrimal sac NHL is a rare malignancy originating in the lymphoid tissue of the lacrimal drainage system. Diagnosis often involves clinical evaluation, imaging studies and tissue biopsy. Treatment options may include surgery, chemotherapy, radiotherapy, immunotherapy, targeted therapy or a combination of the aforementioned strategies, depending on the severity and characteristics of the disease. Surgical excision combined with chemotherapy typically yields favorable outcomes, with adjuvant radiotherapy applied in some cases. Advanced imaging techniques aid in accurate disease staging and treatment planning, contributing to improved patient outcomes. Despite advances in research, the diagnosis and treatment of lacrimal sac NHL remains challenging due to the rarity of the disease. Thus, multidisciplinary approaches are required. Further research into the molecular mechanisms underlying lacrimal sac NHL and potential targeted therapies is crucial for refining clinical strategies. Further understanding of treatment efficacy and the importance of individualized approaches tailored to patient characteristics and disease stage is critical for improving care and prognosis. Continued efforts and collaborations in this field are essential to enhance diagnostic and therapeutic methods, ultimately leading to improved patient outcomes and quality of life.

## Figures and Tables

**Table I:** Staging classification for NHL: Overview of stage definitions.

Stages	Characteristics
I	Involvement confined to one lymph region or a single extranodal site
II	Presence of the disease in two or more lymph regions on the same side of the diaphragm, possibly with limited contiguous extranodal involvement
III	Involvement in the lymph nodes, spleen, or both, and occurring on both sides of the diaphragm
IV	Extranodal involvement, such as in the bone marrow, lungs, or liver

NHL, non-Hodgkin's lymphoma.

**Table II:** Symptoms of NHL involving the lacrimal sac, presenting the most commonly observed symptoms first, progressing to those seen in more advanced stages.

Symptoms
Epiphora
Persistent edema or a mass in the medial canthal region
Redness or irritation of the eye
Discharge from the inner corner of the eye (clear, mucoid, or purulent)
Recurrent eye infections
Pain or discomfort around the eye
Eyelid ptosis
Decreased visual accuracy
Diplopia/visual disturbances
Proptosis/displacement of the eye
Retrobulbar resistance
Chemosis
Nasal symptoms (sinusitis, nasal obstruction, epistaxis)

NHL, non-Hodgkin's lymphoma.

## Data Availability

Not applicable.
